# Evaluation of Zeolite Composites by IR and NMR Spectroscopy

**DOI:** 10.3390/molecules29184450

**Published:** 2024-09-19

**Authors:** Francesco Dalena, Eddy Dib, Barbara Onida, Giorgia Ferrarelli, Marco Daturi, Girolamo Giordano, Massimo Migliori, Svetlana Mintova

**Affiliations:** 1 ENSICAEN, UNICAEN, CNRS, Laboratoire Catalyse et Spectrochimie, Normandie University, 14000 Caen, Francesvetlana.mintova@ensicaen.fr (S.M.); 2 Department of Applied Science and Technology, Polytechnic of Turin, 10129 Torino, Italy; barbara.onida@polito.it; 3 Chemical Engineering, Catalysis and Sustainable Processes Laboratory, University of Calabria, 87036 Rende, Italy; giorgia.ferrarelli@unical.it (G.F.); massimo.migliori@unical.it (M.M.)

**Keywords:** acidity, Brønsted acid site, infrared spectroscopy, NMR spectroscopy, zeolite

## Abstract

In this study, we assessed the quantity, strength, and acidity of zeolite composites comprising Silicalite-1 grown on ZSM-5 crystals using a combination of infrared (IR) and solid-state nuclear magnetic resonance (NMR) spectroscopy. The composites were created through the direct growth of Silicalite-1 crystals on ZSM-5 (P_ZSM-5), either with or without the organic structure-directing agent (OSDA) introduced into the ZSM-5 channels (samples: H_ZSM-5_Sil1 and TPA_ZSM-5_Sil1). The results revealed that Silicalite-1 grew differently when the ZSM-5 core was in the H^+^ form (empty pores) compared to when the OSDA was still present in the sample. This distinction was evident in the textural properties, with a decrease in the micropore surface area and an increase in the external surface area in the H_ZSM-5_Sil1 compared to the parent sample. The TPA_ZSM-5_Sil1 composite exhibited characteristics similar to the parent zeolite. These findings were further supported by ^29^Si NMR, which revealed a comparable local order for the parent (P_ZSM-5) and TPA_ZSM-5_Sil1 samples, along with a broadening of the Q^4^ peak for the H_ZSM-5_Sil1 composite. Additionally, the acid sites were preserved in the TPA_ZSM-5_Sil1 composite, while in the H^+^-form core, the concentration of Brønsted acid sites significantly decreased. This reduction in isolated Brønsted acid sites was further corroborated by ^1^H NMR.

## 1. Introduction

Hydrophobicity and acidity play a determining role on the properties and lifetime of zeolites used either as catalysts or adsorbents in the chemical industry [1]. These features are controlled by the number and location of heteroatoms (e.g., aluminum) in the zeolite framework. The location of these sites and their distribution on specific tetrahedral sites are also crucial in determining the selectivity and activity of the materials [2]. Several spectroscopic methods involving nuclear magnetic resonance (NMR) [3,4], Fourier transform infrared (FTIR) spectroscopy [5] and UV/Vis spectroscopy [6], or theoretical calculations [7] have been used to study the distribution of Al in zeolites, a continuously challenging task nowadays. On another scale, the distribution of active sites either on the surface or in the porosity plays a major role in the application of zeolites. It is then of outmost importance to investigate the differences and distribution of potential active sites and defects between the external surface and the pores of zeolites known for their shape selectivity [8]. This is one of the reasons behind the development of hierarchical porous materials, aiming to reduce diffusion limitations and control the accessibility of acid sites [9,10]. These objectives are also reached through the preparation of composite materials aiming to combine the porosities of two zeolites or to passivate the surface of a zeolite to enhance their catalytic performances by eliminating surface reactions favoring the shape selectivity within micropores. Several post-synthetic approaches have been developed to prepare composite materials with variable surface and bulk properties [11,12,13]. However, surface passivation was shown to induce the blockage of pore mouths, which resulted in reduced catalyst activity. An alternative approach involves the growth of a ‘shell’ on ‘core’ crystallites generating structures wherein the core and shell are of distinct compositions or crystalline structures [14]. Other approaches for composite materials combining zeolites and mesoporous materials aiming to connect different porous networks (i.e., micropores and mesopores) with enhanced stability and diffusion properties were developed [15,16]. Several works targeting the zeolitization of mesoporous supports by the partial conversion of their walls into a zeolite deposit using hydrothermal approaches have also been reported [17]. In the materials obtained, access to the porosity and preservation of the acidity are still questionable and require further improvement. In this work, we report on the synthesis of composites following two pathways. In the first, the growth of Silicalite-1 on ZSM-5 was conducted after calcination (without OSDA) while in the second, the as synthesized ZSM-5 (containing the OSDA) was subjected to Silicalite-1 growth prior to calcination. The acidity and defect sites of the composites were evaluated by combining FTIR and NMR spectroscopy.

## 2. Results and Discussion

### 2.1. Textural Properties of Composites

Two Silicalite-1/ZSM-5 composite samples (TPA_ZSM-5_Sil1 and H_ ZSM-5_Sil1) were prepared from the parent sample ZSM-5 and named P_ZSM-5, according to the procedure described in the Materials and Methods section. The XRD patterns of these samples (Figure S1) confirmed the purity of the crystalline MFI phase; only the characteristic peaks of the MFI type structure were detected for all samples. Moreover, no evident peaks related to amorphous silica were identified. In addition, from the Bragg peaks in the range 23–25 2 Theta degree, it is evident that the orthorhombic phase of ZSM-5 was obtained. The Si/Al ratios and the textural properties of the samples determined by N_2_ physisorption measurements are summarized in Table 1.

**Figure 1:**
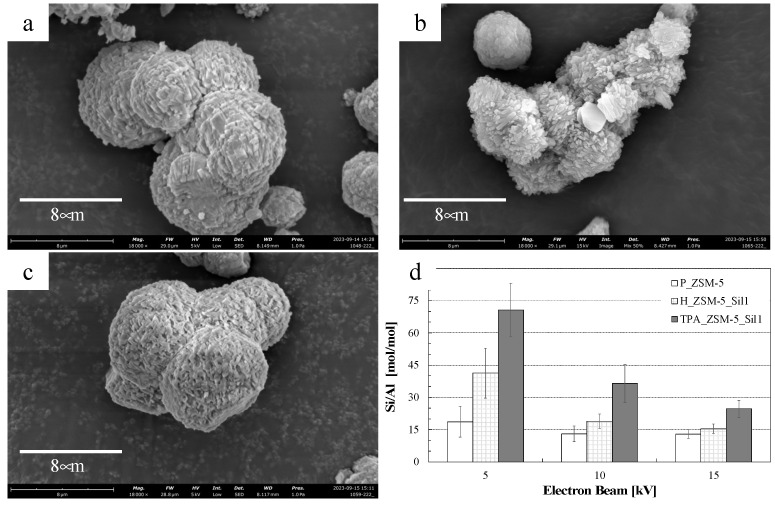
SEM images of samples (**a**) P_ZSM-5; (**b**) H_ZSM-5_Sil1; (**c**) TPA_ZSM-5_Sil1, and (**d**) Si/Al ratio of the samples measured by EDX at different electron beam voltages.

The increase in the Si/Al ratio for the TPA_ZSM-5_Sil1 and H_ZSM-5_Sil1 composites confirmed the successful deposition of Silicalite-1 on the ZSM-5 core samples. Comparing the composites with the parent ZSM-5 zeolite, rises of 10 and 15% in the Si content were observed for the H_ZSM-5_Sil1 and TPA_ZSM-5_Sil1 composites, respectively. Furthermore, although the TPA_ZSM-5_Sil1 sample exhibited a higher Si/Al ratio than the starting zeolite, its total acidity was not substantially lower. SEM micrographs of the samples are presented in Figure 1. The results suggest that the presence of OSDA in the ZSM-5 core sample (TPA_SM-5_Sil1) promoted the growth of Silicalite-1; the ultimate crystal morphology of the parent sample was preserved. In addition, EDX measurements (Figure 1d) performed at different electron beam voltages (namely 5 kV, 10 kV, and 15 kV) showed a decrease in the Si/Al ratio when the beam penetration increased from 10 kV to 15 kV. In addition, sample TPA_ZSM-5_Sil1 exhibited a higher Si/Al ratio than that of H_ZSM-5_Sil1. Therefore, the ZSM-5 zeolite core containing OSDA promoted the homogeneous growth of Silicallite-1.

No significant difference in the specific surface area for the three samples was measured, while some changes in the micropore volume and external surface area were found. The TPA_ZSM-5_Sil1 composite exhibited a slight increase in the micropore area and the micropore volume, while the H_ZSM-5_Sil1 composite showed the opposite behavior, where a 10% lower micropore area was measured. The changes of the external surface area followed a different trend: about a 26% higher external surface area for the H_ZSM-5_Sil1 composite compared to the parent zeolite was measured, while the TPA_ZSM-5_Sil1 was similar to the parent P_ZSM-5 sample. The decrease in the micropore area for the H_ZSM-5_Sil1 sample may have been due to the deposition of Silicalite-1 crystals at the mouth of the empty micropores of the starting acidic zeolite core. The increase in the external surface area implies a disordered growth of Silicalite-1 over the parent zeolite surface. However, the presence of the organic template in the core of the TPA_ZSM-5_Sil1 composite stimulated the growth of ordered Silicalite-1 crystals on the surface, leading to a higher micropore area.

### 2.2. FTIR Characterization of Composites

**Figure 2:**
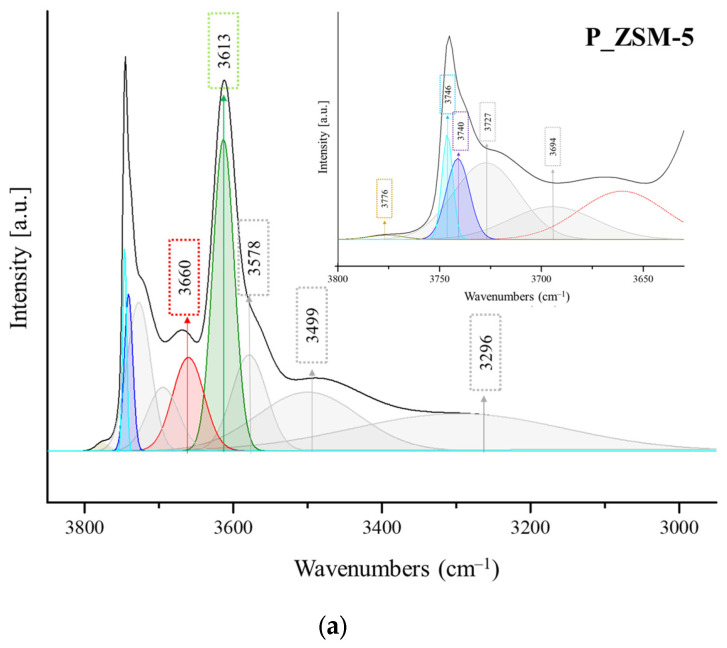

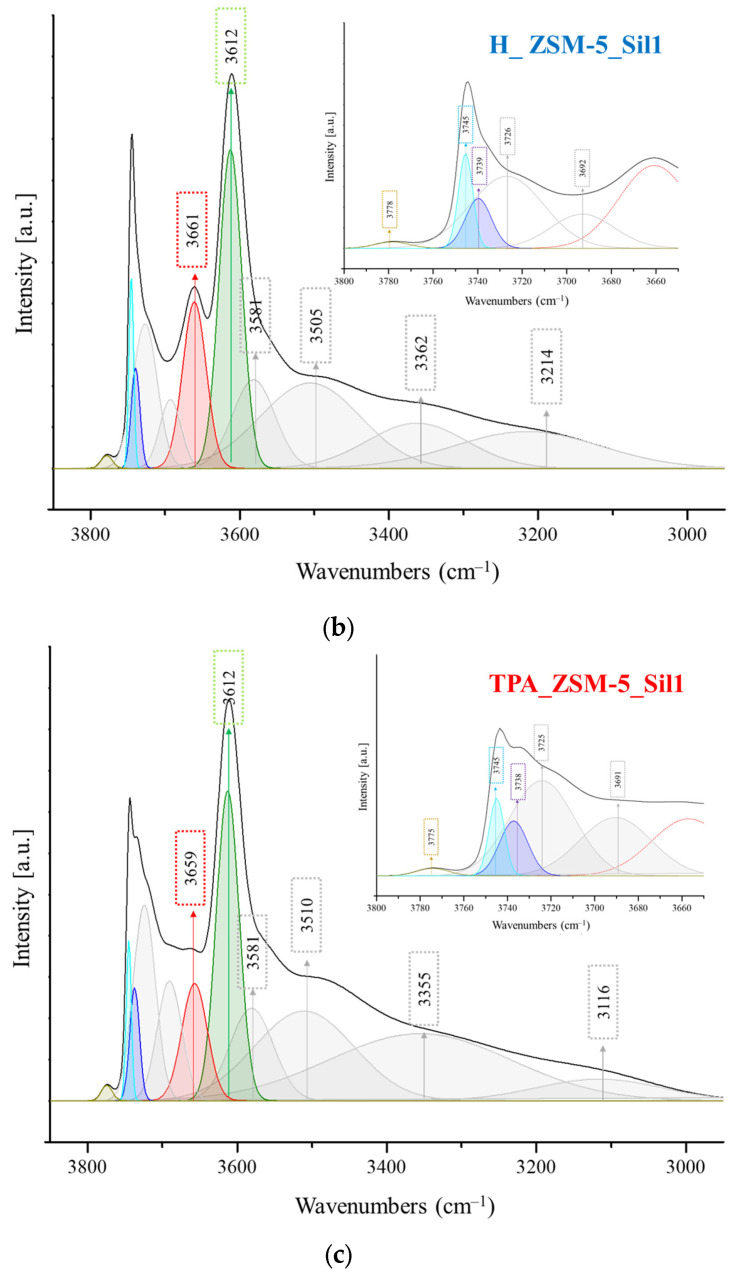
FTIR spectra of the activated P_ZSM-5 (**a**), H_ZSM-5_Sil1 (**b**), and TPA_ZSM-5_Sil1 (**c**) samples in the region 3900–3200 cm^−1^ with the respective deconvolution curves. Inset: Spectra presented in the range of 3600–3800 cm^−1^.

The FTIR spectra of samples in the region of the ν_O−H_ vibrations are depicted in Figure 2. The different absorbance values of the ν_O−H_ bands vary depending on the relative abundance of each functional group. The bands corresponding to the hydroxyl stretching modes in the range of 3750–3735 cm^−1^ indicate the presence of isolated (free) SiOH groups [18,19,20,21,22]. These peaks correspond to two different types of isolated (free) SiOH groups that are not involved in hydrogen bonding: the absorption band at 3745 cm^−1^ is usually ascribed to isolated silanols located on external surface of the crystals, while the band at 3740 cm^−1^ (Figure 2, Scheme S1a) is assigned to the stretching of the hydroxyl groups located inside the zeolite channels [7,23]. The adjacent silanols interact with each other via hydrogen bonding and are associated with broad bands at a low frequency due to the H-bonding donor character in the range of 3650–3500 cm^−1^ [20], while the bands located in the range of 3730–3700 cm^−1^ are related to the H-bonding acceptors, and these species are considered as weak Brønsted acid sites (BAS). In all of the spectra, an intense band at 3610 cm^−1^ was observed, which was assigned to the bridged hydroxyl groups (AlOHSi) that corresponded to the strong Brønsted sites (Scheme S1b), while the extra framework Al−OH species were observed at ca. 3680−3660 cm^−1^ (Scheme S1c). An overlapping of bands due to both the extra framework Al−OH and weakly interacting vicinal OH groups was expected [24].

The broad bands in the region of 3580–3100 cm^−1^ were attributed to the bridged Si−O(H)−Al and silanol Si−OH groups perturbed by a strong H-bonding. No big difference in the Si−O(H)−Al sites was found for the three samples, but the three samples differed substantially in the silanol region. Both composites H_ZSM-5_Sil1 and TPA_ZSM-5_Sil1 exhibited higher absorption in the 3850–3100 cm^−1^ region than the parent sample P_ZSM-5. In addition, in the absorption region of 3730–3700 cm^−1^, the TPA_ZSM-5_Sil1 composite showed a greater absorption. Instead, the H_ZSM-5_Sil1 composite showed a higher absorption in the region of 3680–3660 cm^−1^. This difference in absorption can be related either to the contribution of the extra framework aluminum or to a significant amount of weakly interacting silanols.

### 2.3. NMR Study of Composites

**Figure 3:**
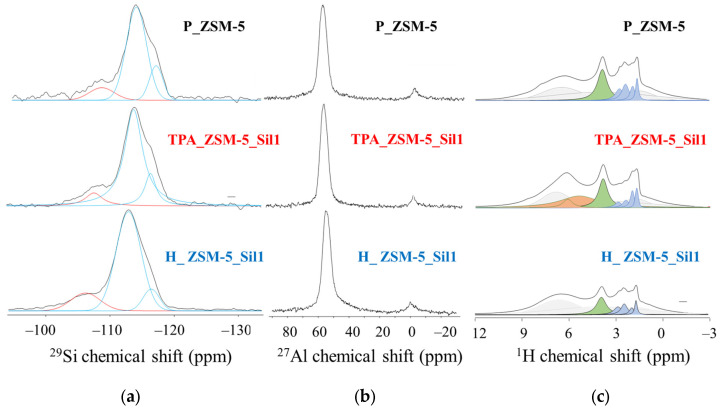
(**a**) ^29^Si NMR spectra, blue lines correspond to Q4 sites, red lines correspond to Q^3^ sites, (**b**) ^27^Al NMR spectra, (**c**) ^1^H NMR spectra, blue lines correspond to isolated and weakly H-bonded silanols, green lines correspond to isolated BAS while orange lines correspond to strongly H-bonded silanols and BAS, grey lines are due to 1.9 mm rotor caps. All samples were pretreated at 623 K under high vacuum (10^−6^ kPa) and the rotors were closed in a glove box under Ar before the acquisition of the spectra. The intensity of the rotor signal (slightly shifting due to differences in magnetic susceptibilities of the samples) was kept constant during the fitting procedure for all samples as in reference [3].

The ^29^Si NMR spectra of the three samples are depicted in Figure 3a. The parent sample P_ZSM-5 and the TPA_ZSM-5_Sil1 composite kept a similar overall local order while a slight broadening in the Q^4^(^0^Al) region (below −110 ppm) was observed for the H_ZSM-5_Sil1 composite (Figure 3a). The Q^4^(1Al) sites (−105 ppm) were assigned to Si-O-Al linkages [25]. The ^27^Al NMR spectra showed the same trend (Figure 3b): a broadening in the peak corresponding to tetrahedral Al sites was observed for the H_ZSM-5_Sil1 composite, while both the P_ZSM-5 and TPA_ZSM-5_Sil1 samples presented a similar trend; a small amount of extra-framework Al was present in all samples [3]. To further understand the local order, high resolution ^1^H NMR spectra were acquired and are shown in Figure 3c. The intensity of the peak at 3.7 ppm, assigned to isolated BAS, decreased by 40% for the H_ZSM-5_Sil1 composite compared to the P_ZSM-5 parent sample, while it only decreased by 4% for the TPA_ZSM-5_Sil1 composite. In addition, broad bands appeared at high chemical shifts above 5.8 ppm in the spectrum of sample TPA_ZSM-5_Sil1, which were assigned to H bonded silanols/BAS [26,27].

### 2.4. Evaluation of Acidity of Composites

The basic probe pyridine (Py) was used to evaluate the acid sites of the composites in comparison to the parent sample (Figure S2). The interaction of Py with both BAS and Lewis acid sites (LAS) in protonated (i.e., pyridinium ion (PyH^+^) and neutral (Py)) forms, were consecutively evaluated [28,29,30]. The Py bonded to the aprotic sites (PyL) showed the characteristic 19b and 8a stretching bands at 1455 cm^−1^ and 1625 cm^−1^, respectively, according to the nomenclature of Kline and Turkevich [31]. In the case of pyridinium ions (PyH^+^), typical modes 19b and 8a at about 1545 cm^−1^ and 1635 cm^−1^ were observed [32]. The integrated area of the 8a and 19b bands increased with the increase in the Py adsorbed on the samples (Figure S2a–c), which corresponded to a concomitant decrease in the OH stretching bands. Starting from the first addition of Py (about 0.2 µmol), all bands decreased, showing that Py did not selectively interact with specific OH groups. Then, the adsorbed Py was removed, first under vacuum treatment at room temperature, and subsequently by heating the samples at 150 °C in vacuum conditions (10^−6^ torr) in order to remove the weakly interacting molecules such as physiosorbed and H-bonded Py. The amount of Py was calculated using the integrated area of the bands and subsequently related to the molar absorption coefficients (Table 2) [33,34].

**Figure 4:**
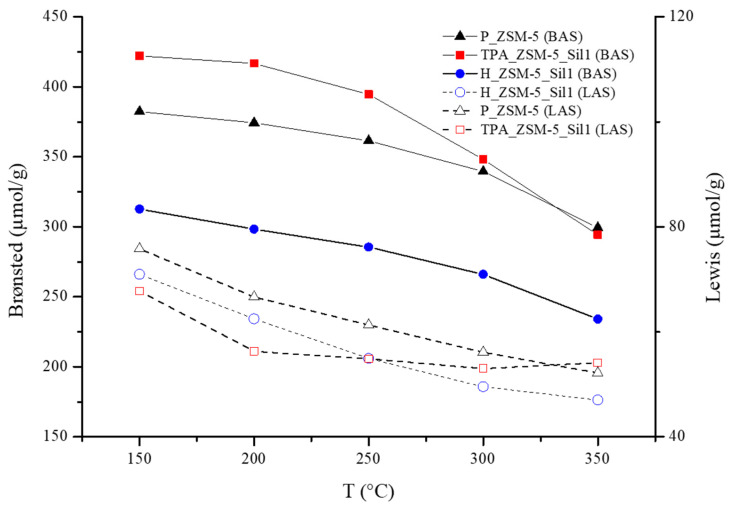
Desorption of Py from Brønsted (solid lines) and Lewis (dash lines) acid sites of samples P_ZSM-5 (black), H_ZSM-5_Sil1 (blue), and TPA_ZSM-5_Sil1 (red) measured between 150 and 350 °C with a step of 50 °C under vacuum (10^−6^ torr).

The linear relationship between absorbance (peak area) and the molar amounts of Py for the three samples was similar to those reported earlier [36]. This may suggest no diffusivity problem in the samples, and consequently, no problem of blocking of the pores, as already demonstrated by the N_2_ adsorption analyses. A slight increase in the amount of BAS for the TPA_ZSM-5_Sil1 composite and a 21% decrease with respect to that of the H_ZSM-5_Sil1 composite were observed, while no significant variation of LAS was measured. This suggests that the crystallization of Silicalite-1 did not lead to an increase in the extra framework Al and that the increase in the peak at 3660 cm^−1^ for the H_ZSM-5_Sil1 composite was due to the increase in some silanol interactions. Furthermore, to evaluate any differences in terms of strength of the acid sites, a desorption study of Py was performed at controlled temperatures and pressures (150–350 °C, 10^−6^ torr). The fast decrease in the area of the bands at 1597 and 1446 cm^−1^ due to H-bonded Py was observed (Figure 4), while pyridinium tended to be quite stable even at high temperatures. The zones corresponding to the absorption of the PyH^+^ cation measured at 300 °C varied only by 22.3, 21.4, and 17.5% for the samples P_ZSM-5, H_ZSM-5_Sil1, and TPA_ZSM-5_Sil1, respectively, compared to the values measured at 150 °C.

### 2.5. Study of Silanol Interactions

**Figure 5:**
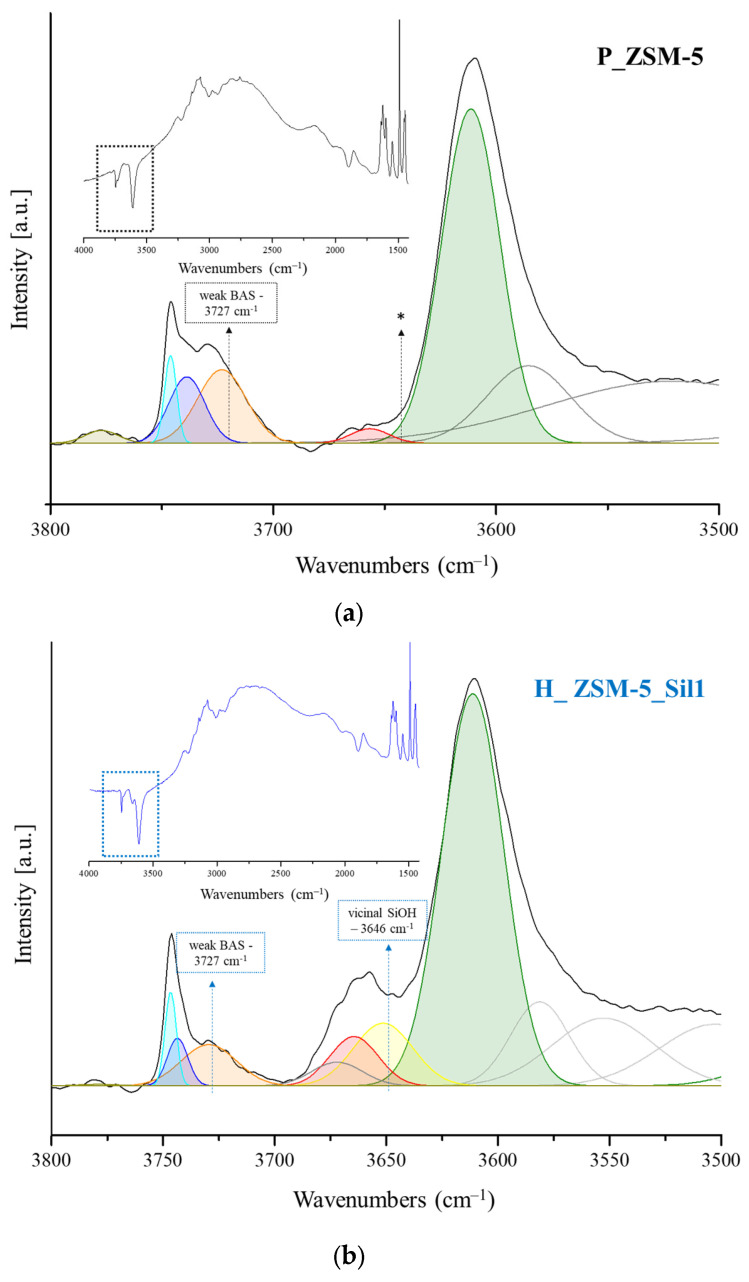

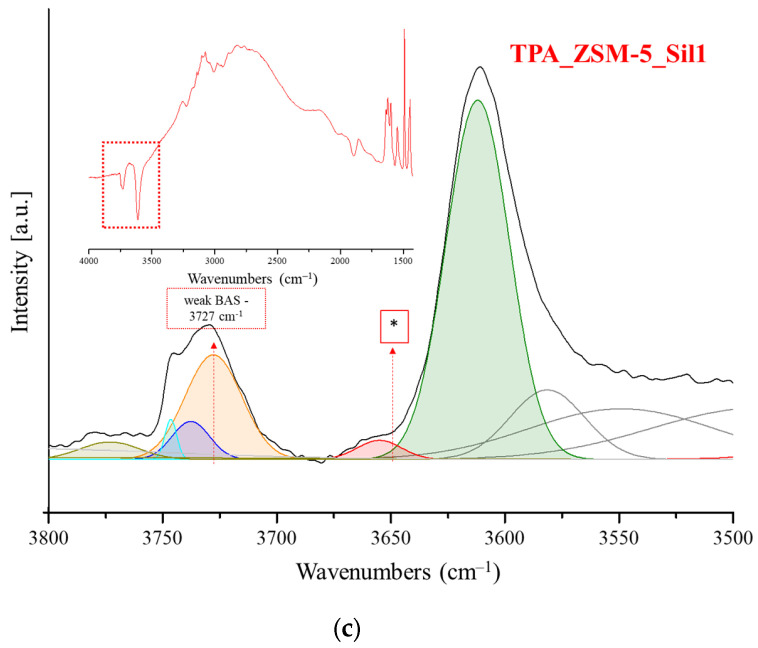
Deconvolution of the FTIR spectra on the OH bands (3800–3500 cm^−1^) of the sample spectra of samples P_ZSM-5 (**a**), H_ZSM-5_Sil1 (**b**), and TPA_ZSM-5_Sil1 (**c**). Spectra represent the differences of the area of the spectra acquired after the adsorption of Py (25 °C, 10^−6^ torr) and the one acquired after the activation procedure (10^−6^ torr at 450 °C for 2 h). Inset: Spectra in the region of 1500–4000 cm^−1^. * The asterisk wants to emphasize the absence of the gaussian function of vicinal silanols (3646 cm^−1^) present in the H_ZSM-5_Sil1 (**b**) sample and not present in the P_ZSM-5 (**a**) sample, nor in the TPA_ZSM-5_Sil1 (**c**) sample.

To verify the co-crystallization of Silicalite-1 on the MFI sample, the evaluation of silanols in the three samples was performed by combining the FTIR and NMR analyses. Additional characterization of the composites consisting of Silicalite-1 grown on ZSM-5 was provided by the semi-quantitative evaluation of the contribution of the free silanols and the silanols interacting via H-bonds. To identify the contribution of these silanols, a peak-fitting of the hydroxyl region was performed. The spectra were obtained by subtracting the one recorded after activation (initial spectra) from the ones recorded at the adsorption of Py at 25 °C. In this case, the area of the bands was proportional to the number of hydroxyls sites occupied by Py at 25 °C (Figure 5).

**Figure 6:**
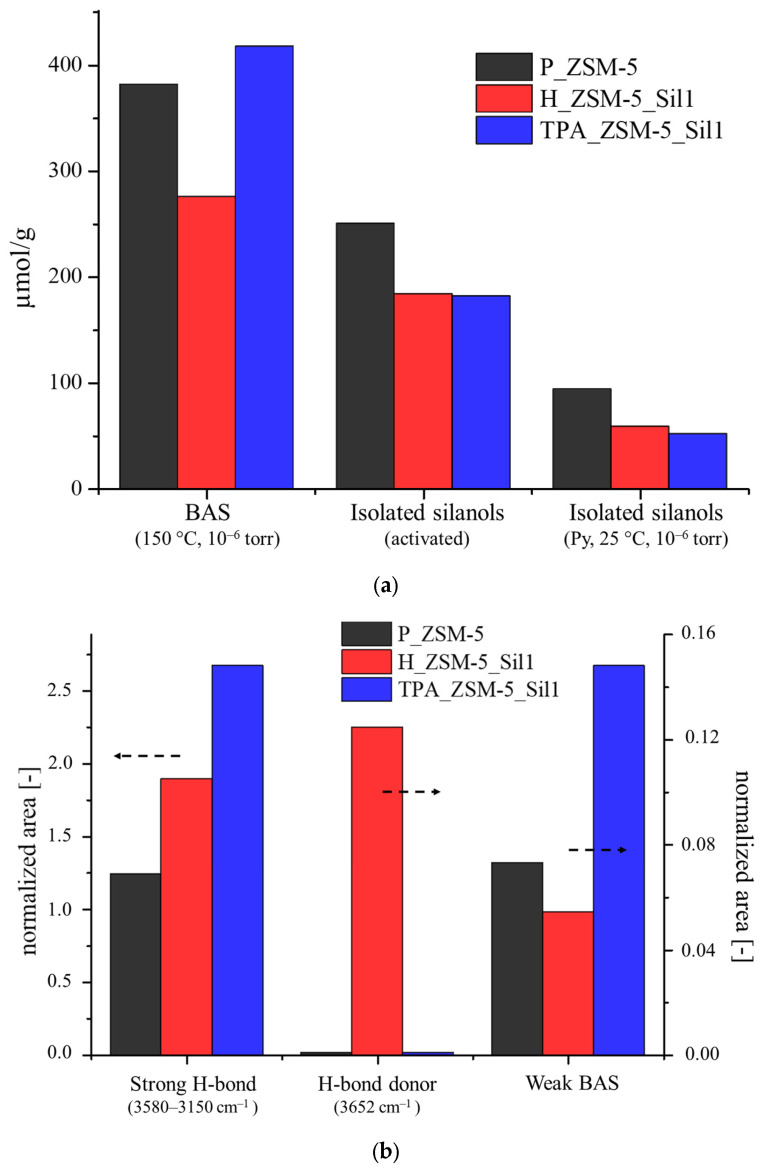
(**a**) Contribution in terms of concentration of BAS, sum of isolated silanols after activation (10^−6^ torr at 450 °C for 2 h), and after the adsorption of Py at 25 °C (Peq = 2.022 torr, 10^−6^ torr). (**b**) Contribution in terms of normalized area for the strong H-bond interactions (3580–3150 cm^−1^), for the donor protons (3652 cm^−1^), and for the weak BAS (3730 cm^−1^) of the samples P_ZSM-5 (black), H_ZSM-5_Sil1 (blue), and TPA_ZSM-5_Sil1 (red).

Aside from the presence of Brønsted and Lewis sites in the samples, the silanols present were sufficiently acidic and may interact with the basic probe at room temperature. Among these silanol species, the free silanols were similar for the three samples, while the contribution of the terminal H-bonded species responsible of the band at 3727 cm^−1^ seemed to be different (Table 2 and Figure 6). This contribution is related to the weak BAS, and in the TPA_ZSM-5_Sil1 composite, they were more than two times higher than in the parent P_ZSM-5 sample, and three times more than in the H_ZSM-5_Sil1 composite. Further deconvoluting the peak at 3660 cm^−1^ for the H_ZSM-5_Sil1 composite clearly showed that there was an overlap of two or more functions (Figure 6b). There was a contribution from the presence of extra framework aluminum, but also a second band located at 3651 cm^−1^, which was tentatively attributed to H-bond donors in the vicinal position. This band was completely absent in both the P_ZSM-5 and in the TPA_ZSM-5_Sil1 samples.

**Figure 7:**
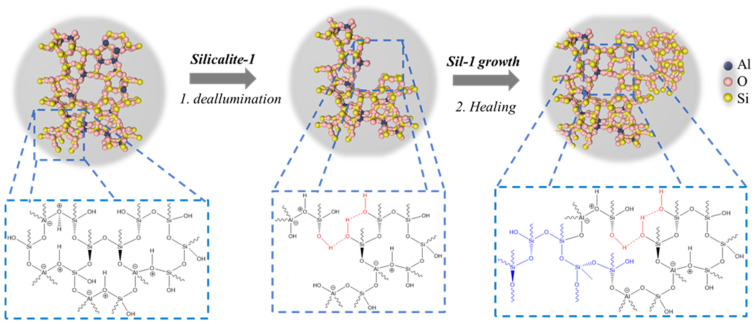
Schematic representation of the growth of Silicalite-1 on the parent P_ZSM-5 resulting in the formation of the composite with a lower amount of silanol defect sites.

The results suggest that in both cases, Silicalite-1 crystallized on the parent zeolite core (P_ZSM-5) either in the as-made or acid form. The chemical compatibility of the parent crystals (P_ZSM-5) and that of Silicalite-1 allowed the crystallization in both samples; a rapid outer Silicalite-1 growth around the zeolite ZSM-5 core was achieved [37]. Furthermore, the dTBPy adsorption data (Table 2 and Figure S3) did not reveal substantial changes in terms of accessibility of the acidic sites of sample H_ZSM5_Sil1. However, it cannot be excluded that the applied hydrothermal treatment during the crystallization of Silicalite-1 did not cause any dissolution of the core ZSM-5 crystals [38]. More specifically, sample H_ZSM-5_Sil1 showed a decrease in Brønsted and Lewis sites of 32% and a 21%, respectively, and the formation of silanol nests (3646 cm^−1^). This was probably due to a partial dealumination of the H_ZSM-5_Sil1 sample along the hydrothermal treatment. The removal of Al from the framework might favor the formation of silanol nests. Once these silanol nests (vacancies) are formed, Silicalite-1 tends to “heal” them, following the mechanism proposed by Yoshioka et al. [39]. In addition, the formation of H-bond-acceptor silanols due to the interactions attributed to the vicinal silanols did not lead to a drastic change in the concentration of the isolated silanols. This effect can be explained by the presence of open pore mouths (the core is in acid form), leading to the growth of Silicalite-1 in a more misaligned manner with respect to the ZSM-5 core, thus affecting the silanol amount and configuration (sample H_ZSM-5_Sil1, Figure 7). This hypothesis is supported by the porosimetry data, in other words, the Silicalite-1 crystals grew in a more disordered manner on the parent zeolite, leading to an increase in the external surface of about 26% and a decrease in the micropore area compared to the parent sample P_ZSM-5.

Regarding the case study of the TPA_ZSM-5_Sil1 sample, the second crystallization step did not lead to substantial changes, leading to a more ordered structure consisting of Silicalite-1 deposited on the surface of the acid catalyst (ZSM-5) with a higher micropore area. In agreement with the NMR and FTIR data, the hydrothermal treatment in this sample did not lead to a change in the total concentration of the acidic sites, but mainly to the formation of a new species of silanol/BAS identified at 5.8 ppm in the ^1^H NMR spectrum and at a band of 3727 cm^−1^ in the FTIR spectrum, which can be considered as weak Brønsted sites, as already reported by Tarach et al. [40]. The difference in terms of the concentration of BAS and LAS according to the NMR and FTIR was further demonstrated by the formation of weak BAS. According to the ^1^H NMR results, the concentration of BAS remained almost unchanged, while in the FTIR spectrum, a lowering of the Lewis sites and a slight increasing of the Brønsted sites could be seen. This was observed by Trombetta et al. [41] and by Hensen et al. [42], and is related to the formation of sites consisting of silanol groups that can interact with strong Lewis acid sites, thus forming a bridging hydroxyl group in the presence of a base (Scheme S2). The slight numerical discrepancy in the calculation (469 ± 8 µmol/g for the parent sample versus 490 ± 9 µmol/g for the TPA_ZSM-5_Sil1 sample) is likely due to the fact that these sites, with a slightly more distorted geometry, may have a different extinction coefficient compared to classical BAS. In summary, in the H_ZSM-5_Sil1 composite, a less controlled silanol healing by the grown Silicalite-1 was observed, while in the TPA_ZSM-5_Sil1 composite, the Silicalite-1 growth was governed by the organic template present in the core, leading to more pronounce healing of the silanols.

## 3. Materials and Methods

### 3.1. Materials

Two Silicalite-1/ZSM-5 composite samples (TPA_ZSM-5_Sil1 and H_ ZSM-5_Sil1) were prepared from the parent sample (ZSM-5, sample named P_ZSM-5), according to the modified procedure described in the literature [18]. Sample P_ZSM-5 was synthesized using the following precursor gel composition: 1 SiO_2_–0.02 Al_2_O_3_–0.08 Na_2_O–0.08 TPABr–20 H_2_O. The precursor gel was prepared under stirring of tetrapropylammonium cations (TPA) as OSDA, sodium hydroxide (NaOH), aluminum hydroxide (Al(OH)_3_), ultrapure water, and a silica gel precipitated as a source of silica for 2 h at room temperature. After crystallization at 170 °C for 4 days, the as-synthesized parent zeolite was used for the preparation of sample TPA_ZSM-5_Sil1 following the procedure described elsewhere [17,18]. In addition, the parent sample was calcined at 550 °C for 6 h in a tubular oven, then ion exchanged twice with a 1 M solution of NH_4_Cl at 80 °C for 2 h, followed by a second calcination at 500 °C (6 h) and used for the preparation of sample H_ ZSM-5_Sil1. Silicalite-1 was synthesized using a precursor mixture with the following molar composition: 2SiO_2_–0.5TPAOH–8EtOH–120H_2_O. The crystallization of Silicalite-1 on the ZSM-5 samples was carried out under hydrothermal conditions at 180 °C for 24 h. The synthesis procedure for Silicalite-1 was repeated twice, and the obtained TPA_ZSM-5_Sil1 and H_ZSM-5_Sil1 composites were then calcined in air at 550 °C for 8 h with a heating rate of 2 °C/min.

### 3.2. Characterization

#### 3.2.1. Textural Analysis

The crystalline structure of the samples was characterized by X-ray powder diffraction using a Miniflex600 (Rigaku, Tokyo, Japan) with a scanning rate of 0.05°/min in the range 5–50° 2theta. The adsorption/desorption isotherms of N_2_ at −196 °C were recorded by ASAP2020 Micromeritics; the BET surface area, micropore area, and pore volume of the samples were determined. Atomic absorption analysis of the samples was performed by AA 700 (Analytik Jena GmbH, Jena, Germany). Scanning electron microscopy (SEM) combined with EDX analysis of the samples was performed using a Phenom Pro G6 microscope.

#### 3.2.2. FTIR Analysis

We recorded the FTIR spectra in the mid-IR region (4000–400 cm^−1^) with a Nicolet Nexus FTIR spectrometer at a 4 cm^−1^ optical resolution and 64 scans. The samples were pressed into self-supported disks with a radius of 1.6 cm and a weight of about 20 mg. The disks were outgassed under a vacuum of 10^−6^ torr at 450 °C for 2 h (heating rate of 5 °C/min). The amount of the adsorbed gases was controlled by two pressure transducers, measuring the pressure of the entire line and the gas aliquots in the calibrated volume before admitting it into the cell. The temperature of the sample was monitored during the treatment using a thermocouple inserted into the heater compartment of the cell. Py was used as a basic probe molecule; the concentration of BAS and LAS was estimated by integrating the area of peaks at 1545 cm^−1^ and 1455 cm^−1^, respectively. The probe molecule was adsorbed at room temperature on the activated samples. After the saturation of samples with Py, the excess was subsequently removed by evacuating the samples under vacuum at 10^−6^ torr for 30 min to remove the physiosorbed molecules [30,31]. A spectrum was acquired between 150 and 350 °C at every 50 °C to estimate the strength of the acid sites. Based on the model proposed by Gabrienko et al. [20] on the ZSM-5 zeolite, the deconvolution of the FTIR spectra in the hydroxyl region was performed using a “Peakfit”. In addition, to evaluate the external acidity of the samples, an excess of dTBPy probe molecules was adsorbed at 150 °C, and then the physisorbed dTBPy molecules were subsequently removed under vacuum (10^−6^ torr) at the same temperature.

#### 3.2.3. NMR Analysis

Solid-state NMR spectra were acquired on a 500 MHz (11.7 Tesla) Avance III HD spectrometer using a 4.0 mm OD probe head for the 29Si and 27Al NMR spectra, and a 1.9 mm OD probe head for the ^1^H NMR spectra. The 4.0 mm rotors were spun at 12 kHz, while the 1.9 mm rotors were spun at 40 kHz. One pulse experiment was used for every nucleus, the flip angles were π/3, π/6, π/2 with radiofrequency powers of ~38, 33, 114 kHz, recycle delays of 20, 1, 10 s for ^29^Si, ^27^Al, and ^1^H spectra, respectively. The ^29^Si, ^27^Al, and ^1^H spectra were recorded for 256, 1024, 64 scans, respectively.

## 4. Conclusions

Zeolite composites consisting of Silicalite-1 grown on the ZSM-5 zeolite crystals with and without an organic template were characterized by FTIR and NMR spectroscopy. The concentration and strength of the acid sites were evaluated by FTIR using pyridine as the probe molecule. Differences in terms of both the free silanols and H-bonded silanols were found for the composites. The quantity of Brønsted sites for the H_ZSM-5_Sil1 composite decreased compared to the parent P_ZSM-5 sample, while for the TPA_ZSM-5_Sil1 composite, the number of Brønsted sites increased slightly as a function of an increase in the H-bonded silanol acceptor sites. This was due to the different contribution of the isolated silanols, which turned out to be decidedly changed with a number of isolated silanols (3740 cm^−1^) capable of adsorbing Py at 25 °C, which appeared to be about 3 times higher than in the parent sample (P_ZSM-5). When the organic template was absent from the channels of the parent zeolite (ZSM-5), the silicalite-1 grew unevenly, leading to a reduction in Brønsted acid sites of the composite. Conversely, when the organic template was present within the channels of the ZSM-5, it helped maintain the framework structure, promoting a more uniform growth of Silicalite-1. Achieving a consistent Silicalite-1 coating on the surface resulted in samples with a hydrophobic surface that retained the ZSM-5 acidic properties. The absence of acid sites on the surface of the composite is expected to enhance the catalytic performance of the passivated catalysts by eliminating surface reactions that could otherwise affect selectivity within the micropores.

## Figures and Tables

**Table 1:** Textural properties of the parent and composite samples.

Sample	Si/Al ^1^ (mol/mol)	S_BET_ ^2^ (m^2^/g)	S_micro_ ^3^ (m^2^/g)	S_ext_ ^4^ (m^2^/g)
P_ZSM-5	28	384	255	130
H_ZSM-5_Sil1	30	393	229	164
TPA_ZSM-5_Sil1	32	384	264	121

^1^ Calculated by the atomic adsorption technique. ^2^ Calculated by the multipoint BET method in the Rouquerol p/p0 range. ^3^ Calculated by the t-plot method. ^4^ Calculated at p/p0 0.95.

**Table 2:** Acidity of samples measured by the adsorption of Py and 2,6-di-tert-butylpyridine (dTBPy) probe molecules followed by FTIR spectroscopy.

Sample	Acid Sites			Silanols			External Acidity (dTBPy)	
	BAS		LAS	Isolated Silanols (3745–3740 cm^−1^)		Weak BAS (Normalized Area) ^6^ [–]	dTBPyH^+ 7^ [µmol/g]	Accessibility Factor (PyH^+^/dTBPyH^+^)
	PyH^+ 1^ [µmol/g]	Isolated ^2^ Si−O(H)−Al [µmol/g]	PyL ^3^ [µmol/g]	Activated ^4^ [µmol/g]	Py (25 °C) ^5^ [µmol/g]			
P_ZSM-5	382 ± 5	330	87 ± 3	251	94	1.63	9	0.02
H_ZSM-5_Sil1	312 ± 3	222	71 ± 5	184	59	1.3	13	0.04
TPA_ZSM-5_Sil1	422 ± 4	353	68 ± 5	182	52	3.06	13	0.03

^1^ Calculated using the IMEC of 1.09 for BAS calculated on ZSM5 (Si-Al = 27–40) of the peak of the Py adsorbed on BAS at 1545 cm^−1^ of the spectra acquired at 150 °C [23]; the standard deviation was calculated from the average of measurements performed in three independent replica. ^2^ Calculated using the IMEC of 3.06 for BAS calculated on ZSM5 of the peak of the hydroxyl stretching of the isolated Si−O(H)−Al at 3612 cm^−1^ of the spectra acquired after the activation of the sample [34]. ^3^ Calculated using the IMEC of 2.22 of the peak of the Py adsorbed on LAS at 1455 cm^−1^ of the spectra acquired at 150 °C [20]; the standard deviation was calculated from the average of measurements performed in three independent replica [33]. ^4^ Calculated using the IMEC of 1.5 for isolated silanols calculated on ZSM5 of the region of the hydroxyl stretching of the isolated Si−OH at 3745–3740 cm^−1^ of the spectra acquired after the activation of the sample [20]. ^5^ Calculated using the IMEC of 1.5 for isolated silanols calculated on ZSM5 of the region of the hydroxyl stretching of the isolated Si−OH at 3745–3740 cm^−1^ [26] of the spectra resulting from the subtraction of the one acquired after the adsorption of Py (saturation and evacuation at 10^−6^ torr) and the activated one. ^6^ Area of the Gaussian function normalized by the weight of the wafer of the peak at 3730 cm^−1^ of the spectra resulting from the subtraction of the one acquired after the adsorption of Py (saturation and evacuation at 10^−6^ torr) and the activated one. ^7^ Calculated using the IMEC of 5.3 for dTBPyH^+^ at 1615 cm^−1^ of the spectra acquired at 150 °C and evacuated (10^−6^ torr) at the same temperature [35].

## Data Availability

Data are contained within the article.

## Supplementary Materials

The following supporting information can be downloaded at: https://www.mdpi.com/article/10.3390/molecules29184450/s1, Figure S1: XRD patterns of the samples P_ZSM-5, H_ZSM-5_Sil1, and TPA_ZSM-5_Sil1; Scheme S1: Schematic representation of (a) free silanols and different possible structures of the interaction of silanols, (b) bridged hydroxyl groups (AlOHSi), and (c) possible structure of extra-framework aluminum; Figure S2: Absorption of Py on samples P_ZSM-5 (a), H_ZSM-5_Sil1 (c), and TPA_ZSM-5_Sil1 (e) performed at different dosing. Desorption profile of Py for samples P_ZSM-5 (b), H_ZSM-5_Sil1 (d), and TPA_ZSM-5_Sil1 (f) in the range of 1700–1350 cm^−1^. Equilibrium pressure = 2.022 torr.; Figure S3. Comparison between the normalized FT-IR spectra of the peak at 1615 cm^−1^ related to dTBPyH^+^ (a). FT-IR spectra subtraction between the spectra of the dTBPyH^+^ at 150 °C and the activated spectra for the samples P_ZSM-5 (b), H_ZSM-5_Sil1 (c) and TPA_ZSM-5_Sil1 (d); Scheme S2. Formation of a bridging hydroxyl group in the presence of pyridine.
