# Supplementary material for: Evaluation of Zeolite Composites by IR and NMR Spectroscopy
Source: Molecules. 2024 Sep 19;29(18):4450. doi: 10.3390/molecules29184450 (PMC11433990; doi:10.3390/molecules29184450)
Supplement: Supplementary file 1 [file molecules-29-04450-s001.zip › molecules-3188055-supplementary.pdf]

molecules

MDPI

Supporting Information

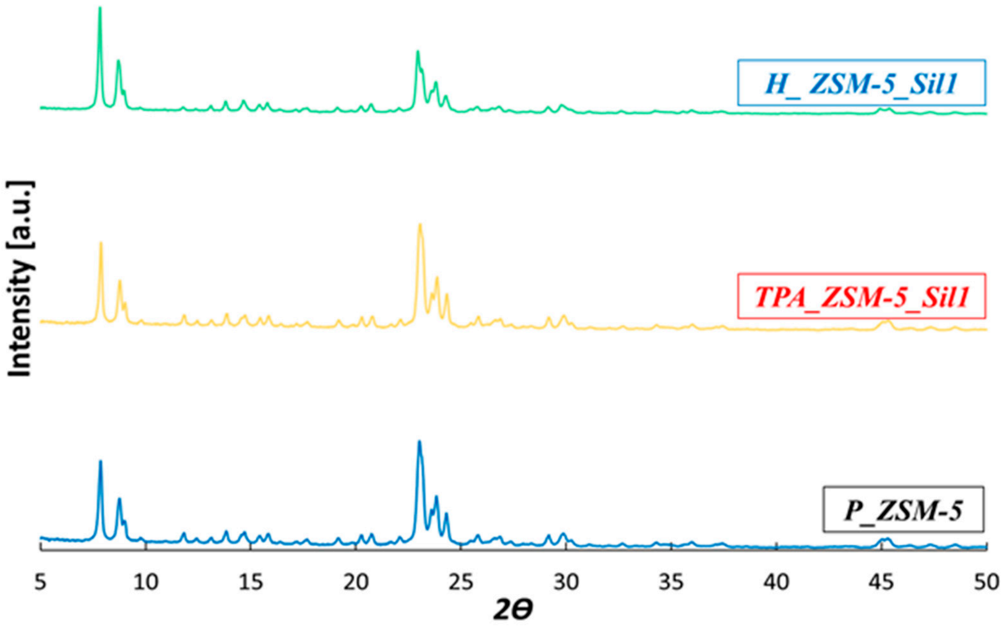

Figure S1. XRD patterns of the sample P_ZSM-5, H_ZSM-5_Sil1 and TPA_ZSM-5_Sil1.

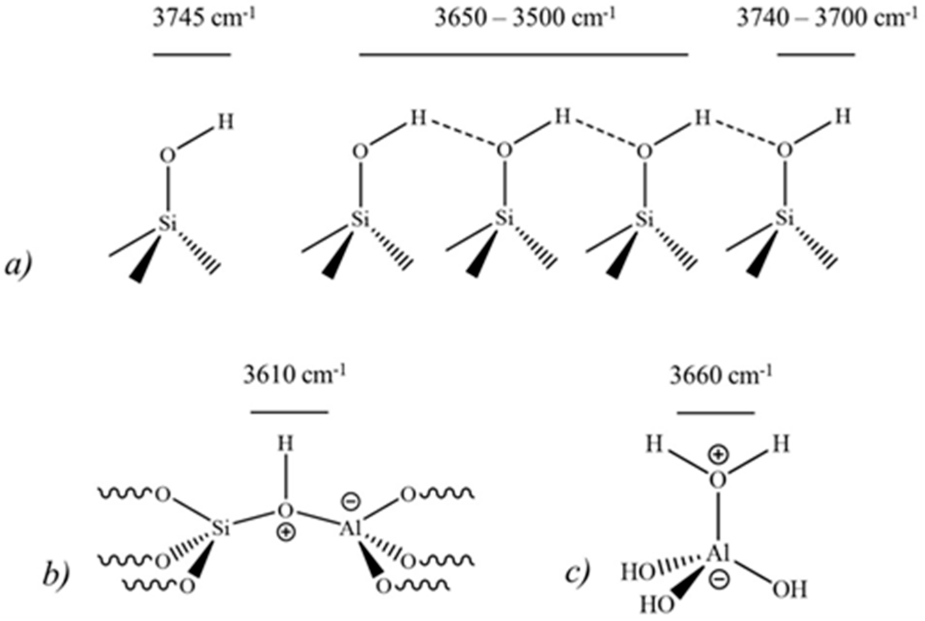

Scheme S1. Schematic representation of (a) free silanols and different possible structures of interacting of silanols (b) bridged hydroxyl groups (AlOHSi) and (c) possible structure of extra-framework aluminum

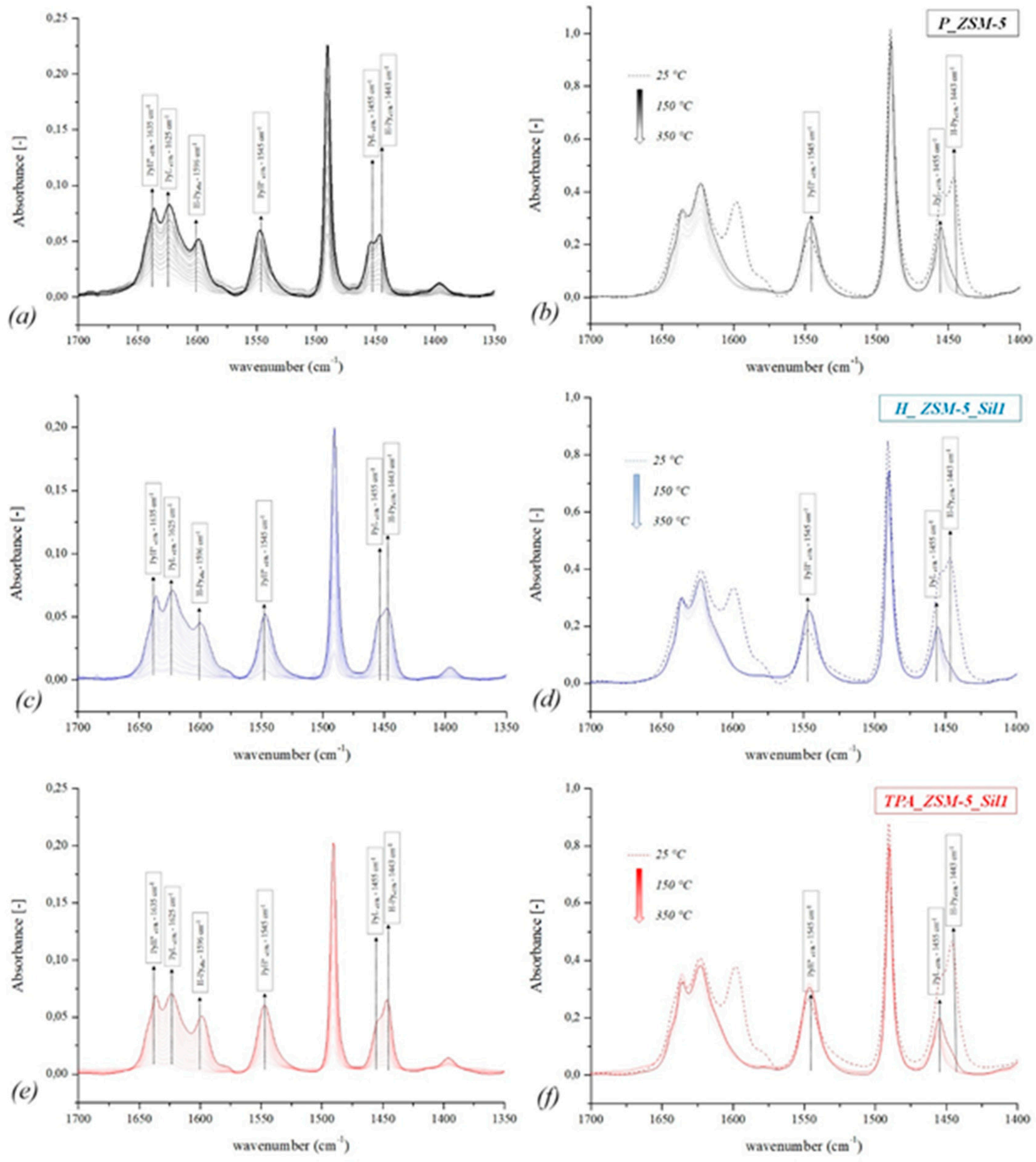

Figure S2. Absorption of Py on samples P_ZSM-5 (a), H_ZSM-5_Sil1 (c) and TPA_ZSM-5_Sil1 (e) performed at different dosing. Desorption profile of Py for samples P_ZSM-5 (b), H_ZSM-5_Sil1 (d) and TPA_ZSM-5_Sil1 (f) in the range of 1700 – 1350 cm^{-1}. Equilibrium pressure = 2.022 torr.

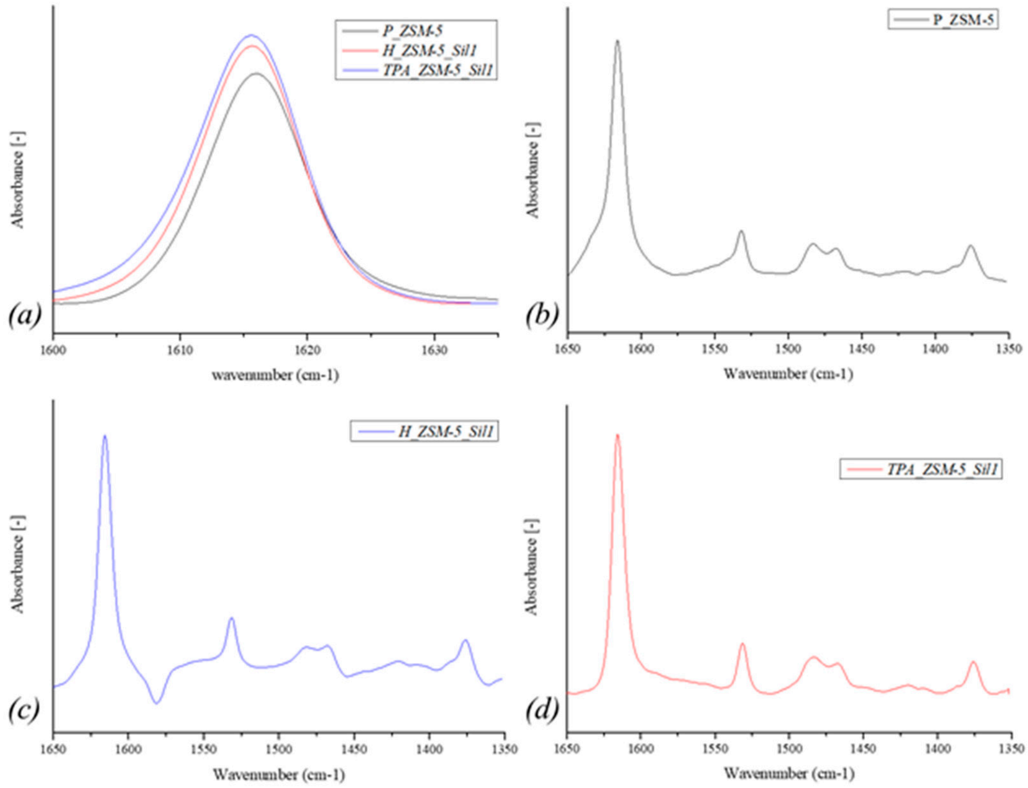

Figure S3. Comparison between the normalized FT-IR spectra of the peak at 1615 cm^{-1} related to $dTBPyH^+$ (a). FT-IR spectra subtraction between the spectra of the $dTBPyH^+$ at 150 °C and the activated spectra for the samples P_ZSM-5 (b), H_ZSM-5_Sil1 (c) and TPA_ZSM-5_Sil1 (d).

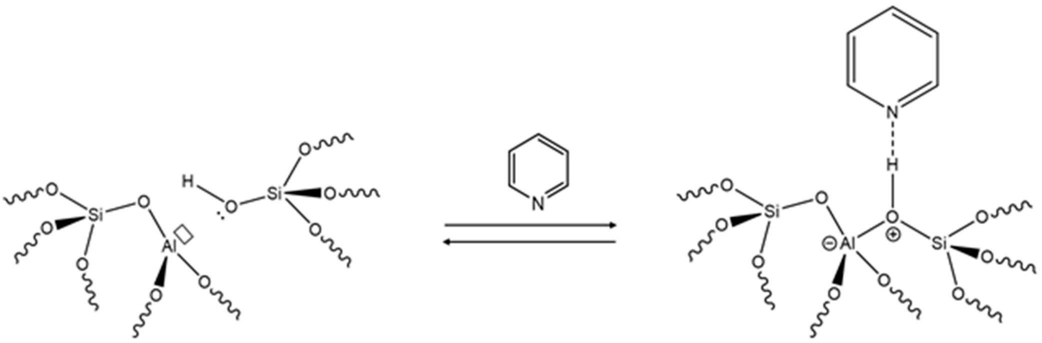

Scheme S2. Formation of a Bridging Hydroxyl Group in the presence of Pyridine
